# The Evolution and Future Trends of Unsaturated Polyester Biocomposites: A Bibliometric Analysis

**DOI:** 10.3390/polym15132970

**Published:** 2023-07-06

**Authors:** Piedad Gañán, Jaime Barajas, Robin Zuluaga, Cristina Castro, Daniel Marín, Agnieszka Tercjak, Daniel H. Builes

**Affiliations:** 1 Facultad de Ingeniería Química, Universidad Pontificia Bolivariana, Circular 1, 70-01, Medellín 050031, Colombia; cristina.castro@upb.edu.co (C.C.); danielbuiles@hotmail.com (D.H.B.); 2 Dirección de Planeación, Unidad de Analítica y Estudios de Contexto, Universidad Pontificia Bolivariana, Circular 1, No 70-01, Medellín 050031, Colombia; jaimealejandro.barajas@upb.edu.co; 3 Facultad de Ingeniería Agroindustrial, Universidad Pontificia Bolivariana, Circular 1, 70-01, Medellín 050031, Colombia; robin.zuluaga@upb.edu.co; 4 ‘Research and Development Center, Andercol-Akzonobel’, Andercol S.A.S, Autopista Norte, 95-84, Medellín 050031, Colombia; 5 ‘Materials + Technologies’ Group (GMT), Department of Chemical and Environmental Engineering, Faculty of Engineering Gipuzkoa, University of the Basque Country (UPV/EHU), Plaza Europa 1, 20018 Donostia-San Sebastian, Spain; agnieszka.tercjaks@ehu.eus

**Keywords:** unsaturated polyester, composites, natural fibers, biocomposites, bibliometric analysis

## Abstract

Unsaturated polyester resin (UPR) is one of the first commercialized polymer matrices for composites reinforced with glass fibers, but has remained popular to this day. To reduce their environmental impact, natural fibers have been used as reinforcements. Researchers all over the world are still interested in these composites, and numerous papers have been published in the last four decades. Using bibliometric analysis, this work provides compiled, structured, and relevant information about the evolution and current state of these materials. This first study on UPR biocomposites based on bibliometric analysis examined 531 published papers identified in the Scopus database from 1982 to July 2022. An analysis of the most active states, leading institutions, and leading authors is followed by the identification of key areas such as the most common natural fibers used as reinforcements, fiber treatments, and composite design parameters such as processing techniques; recently, composite testing; and technological applications. The findings emphasize the importance of staying active in this global field and provide information on novel promising topics for future research.

## 1. Introduction

For decades, unsaturated polyester resins (UPRs) have been used as a basic thermosetting matrix for the fabrication of uncountable composite materials [1]. Thousands of tons of UPR matrices are consumed annually to develop an enormous number of composites, and the global market is expected to grow another 7.1% by 2030 [1]. The main advantages of UPR are its availability at a competitive price worldwide, the vast number of processes including the low-cost of manufacture as in the case of hand lay-up, and the flexible resin-curing process, which can occur at a broad range of temperatures, including room temperature (20 °C and 25 °C) [2].

One of the leading concerns regarding UPR composites is their environmental impact, especially when synthetic reinforced materials such as glass fibers are used. Several strategies have been proposed for reducing this impact. One of the most-documented methods to date is the use of natural fibers [3,4,5]. These composites are part of the recently called biocomposites [6,7].

The widespread use of natural fibers as reinforcements for UPR matrices is based on the many advantages they offer, including low density, recyclability or even biodegradability depending on natural resources, high specific tensile properties, high flexibility, and an abundance of raw materials, some of which are isolated from agro-industrial residues [8,9,10,11]. Furthermore, the separation or extraction of natural fibers requires less energy than the production of synthetic reinforcements such as glass fibers [12].

The growing recognition of UPR biocomposites derives from the widespread availability of raw materials and composite-processing techniques [13], as well as their increasing use in a wide range of applications [14], in the case of the UPR biocomposites, some of them include construction [12], automotive interior parts [15], and even electronic components [16].

Since the early 1980s, thousands of studies on the creation of UPR biocomposites have been conducted [8]. For example, in 2021, the Scopus database reported 919 documents relating to biocomposites. The vast volume of material includes publications such as that by Bledzki and Gassan [10], which has been mentioned over 5000 times. This scenario suggests that sorting through such a big number of data will be difficult especially considering that UPR biocomposites are frequently mentioned as one of several types of biocomposites. There are fewer papers that focus entirely on UPR biocomposites and thoroughly study their evolution throughout time. This illustrates the need for studies that condense material, reveal gaps, and provide junior researchers with condensed and agile knowledge. The use of analytical tools such as bibliometric analysis may be valuable for this proposal.

The use of tools such as bibliometric analysis supported by mapping is gaining popularity due to the benefits of combining the analysis of scientific publications with a visual presentation that makes it useful to have a general overview of a specific topic, suppling the value of data extracted from documents that include authors, affiliations, representative journals, and by the keyword analysis identification of the evolution of trending topics [17,18,19]. These tools are applied annually to analyze trends in engineering topics, including the topic of composite materials, such as the recent bibliometric study on sustainable polymers [17], geopolymer composites [20], and starch-based plastics [21].

Besides the advantages of the bibliometric study, there are two important limitations of this technique, which correspond to the use of software tools such as VantagePoint, which are only available under commercial licenses, and the lack of experience of researchers in how to conduct the literature review [22]. To address these challenges, a team of professionals with experience in bibliometric and technological files, as in the current work, is desirable.

Recognizing the significance of having a work focusing entirely on UPR biocomposites that give agile structural information throughout time, this work presents a bibliometric study of research trends to contribute to recognized new research topics. This study was designed in five steps [22]: (1) study design, (2) data collection, (3) data processing, (4) data visualization, and (5) interpretation. The final search-query equation utilized for data collection was generated during the study design phase. The documents were discovered in the Scopus database. The data was analyzed using a macro- and microlevel approach with parameters such as the most active state in UPR biocomposite research, leading institutions and authors, as well as highly cited writers and documents. From the information supplied by the author’s keywords, it was possible to remark on the evolution of natural reinforcements, information about the main fiber treatments, aspects linked to the UPR biocomposite design and processing, evolution of the composite behavior evaluation, and documented applications of these composites. The collected information was analyzed using Vantage Point software and VOSviewer, whereas Excel and Origin Pro 11 were used for better data visualization. The final step, which corresponded to the interpretation, required an in-depth review of the chosen documents. The results provided a useful map of the field for identifying new research trends in these UPR biocomposites.

## 2. Materials and Methods

**Figure 1:**
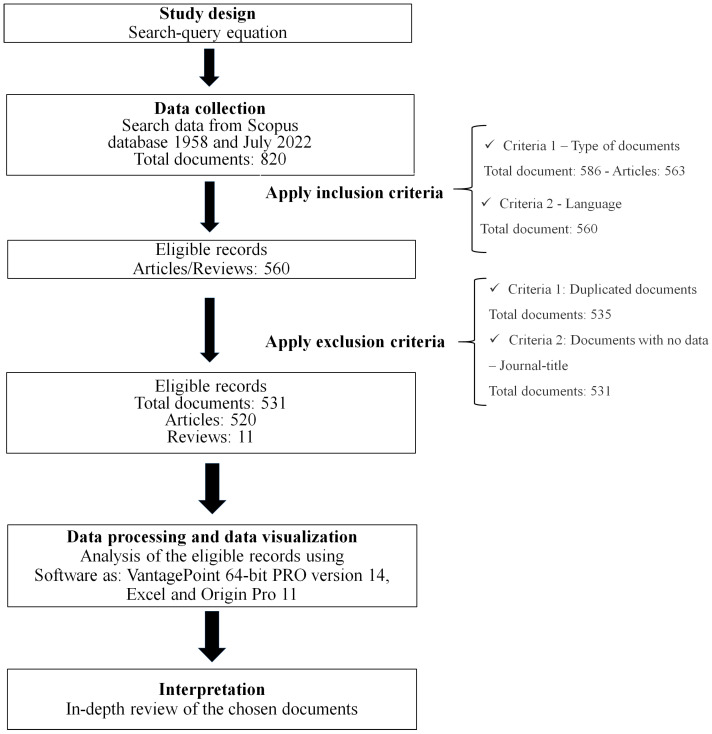
Scheme of the methodology used for bibliometric analysis.

Figure 1 summarizes the methodology used in this study. The first step involves the identification of keywords to find documents denoted as study design. This selection was useful for maximizing the number of useful documents. The records were collected from the Scopus database because it has a broader bibliometric scope and contains more current data than other databases [23,24]. This decision implies a limitation of this study; as Aria and Cuccurullo [22] point out, there is no database that covers all scientific subjects or journals. The usage of supplementary databases such as Scielo or Google Scholar could include works that are not recognized by Scopus, as in the case of a publication without CiteScore. At least five validations were required before creating the final search-query equation. These validations entailed assessing the impact of the obtained papers using keywords associated with different kinds of written word, such as fiber or fibre, and some writers used a variety of phrases to refer to natural fibers. These previous validations revealed the necessity of combining elements such as fib* and cell* with the Boolean operator OR. These components included information on vegetable fibers as well as other natural fibers such as animal fibers and mineral non-synthetic fibers [25]. The data-collecting process allows eligible records to be identified. To exclude documents related to other topics such as ceramic synthetic fibers, works related to the development or characterization of resin or traditional composites, elements associated with biocomposites or green composites such as bio*, nat*, and gre* linked by the Boolean operator OR were used. According to the previous evaluations, the final search-query equation utilized was (“unsat* poly*”) AND (bio* OR nat* OR gre*) AND (fib* OR cell*) AND (comp* OR biocomp*). This equation included in the Scopus corresponds to title, abstract, and keywords (TITLE-ABS-KEY). All documents were collected between 1958 and July 2022, and correspond to the 820 records. As shown in Table 1, most of the 563 identified records were articles.

These texts were written in eight (8) different languages, the majority of which were written in English (95.5%), with Chinese coming in second with 2.1%. There were documents published in languages spoken by fewer than 70 million people worldwide, such as Malay. As shown in Figure 1, two inclusion criteria were used to improve this analysis: (1) type of document: chosen articles and reviews; and (2) language: written in English. Recognizing the importance of meticulously investigating all articles chosen, the two inclusion criteria were employed to ensure that all documents were readily available. After the inclusion criteria were applied, the total number of documents was reduced to 560, comprising 539 articles and 21 reviews. The following phase was an in-depth examination of each document’s TITLE-ABS-KEY to validate the UPR biocomposite theme. Two criteria for exclusion were applied in this analysis: (1) duplicated documents and (2) documents with no data, such as the journal title. After this evaluation, the eligible records used in the bibliometric analysis corresponded to 531 documents: 520 articles and 11 reviews. The Scopus information of the final selected documents was stored in comma-separated values (csv) files. The data-processing and visualization processes were carried out utilizing VantagePoint 64-bit PRO version 14. For additional analyses, software VOSviewer version 1.6.18, Excel, and Origin Pro 11 were used.

The parameters considering a macro- and microlevel approach included parameters such as the most active state in UPR biocomposite research, prominent institutions and authors, and highly cited authors and papers. The data visualization was useful to show the collaborative networks of the co-authors, while the popular keywords used by the authors were useful to show the evolution of the different topics over time, as well as to identify the clusters and thematic areas, as suggested by Cobo et al. [26].

For the interpretation step, the eligible materials were thoroughly examined in order to find the most significant components of the theme categories discovered in the previous steps.

Equations (1) and (2) were used to determine the efficiency of the state contribution to this topic. These equations were proposed in the work of Tanjin Amin et al. (2019) [27]. Information on the population in million per state was obtained from the World Bank website [28]. Equation (3), proposed by Tanjin Amin et al. (2019) [27], was used to evaluate the variation in the publication as a function of state.

(1) $$Publications\;per\;million\;population=\frac{Total\;paper\;by\;a\;state}{Total\;population\;in\;million}$$

(2) $$Publications\;per\;capita\;income\;thousand\;USD=\frac{Total\;paper\;by\;a\;state}{Per\;capita\;income\;per\;thousand\;USD\;}$$

(3) $$Change\;in\;publications=\frac{\left(Publications\;2011-2021-Publications\;before\;2011\right)}{Publications\;before\;2011}\;\times \;100\%$$

## 3. Results and Discussion

**Figure 2:**
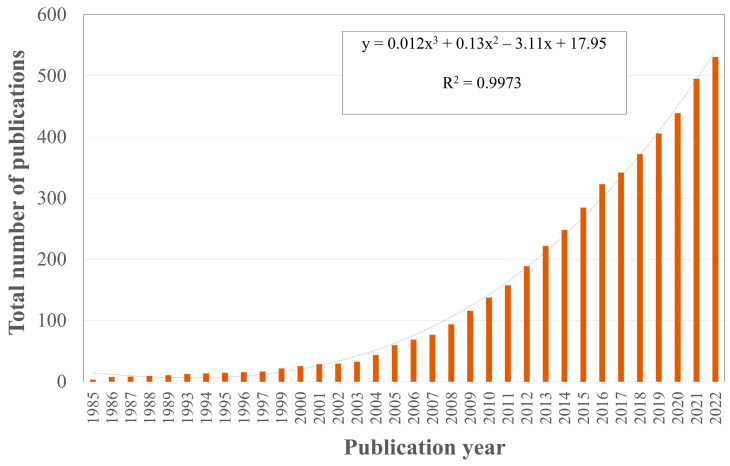
Evolution of the total number of documents.

In this study, 531 eligible papers were analyzed to identify the main trends linked to the evolution of the use of natural resources in UPR biocomposite developments. Analyzing these trends over time is useful for identifying gaps or opportunities in the current literature and proposing future innovations in this growing area. Prior to the detailed analysis, Figure 2 shows the evolution of publications since 1982, when the first study based on the effect of natural fibers as reinforcement for UPR matrices was published by Schaudy and Proksch (1982) [8], which used wood as the natural fiber. This work was published in the Industrial and Engineering Chemistry Product Research and Development. This journal was edited by ACS Publications until 1986.

One remarkable aspect observed in Figure 2 is the continuously increasing number of publications since 1986. Notably, the relevance rose from January 2005 and July 2022. During this period, 91.7% of the identified articles were published, of which 74% were published between 2011 and July 2022, and 67.2% between 2011 and 2021. The total number of publications by year corresponds to the equation y = 0.012x^3^ + 0.13x^2^ − 3.11x + 17.95. This equation demonstrates the increasing importance of publishing. The appropriate R2 value for this outcome is 0.9973. This indicates that UPR biocomposites continue to be a significant and growing issue. In the following subsections, a detailed bibliometric analysis of the development of UPR biocomposites is presented. This study takes a macrolevel approach, identifying and analyzing the impact of states with more active UPR biocomposite research. The microlevel study focused on identifying the institutions and main authors, as well as an examination of the author’s keywords. The examination of the author’s keywords provides information about trend subjects and their evolution over time.

### 3.1. States Active in UPR Composite Research

**Figure 3:**
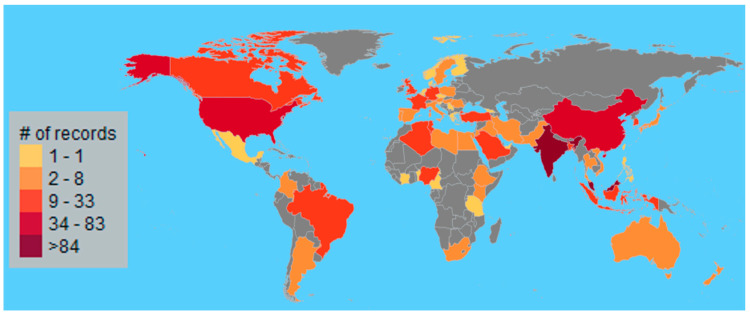
Global distribution of the authors, elaborated based on data obtained using VantagePoint 64-bit PRO version 14.

**Figure 4:**
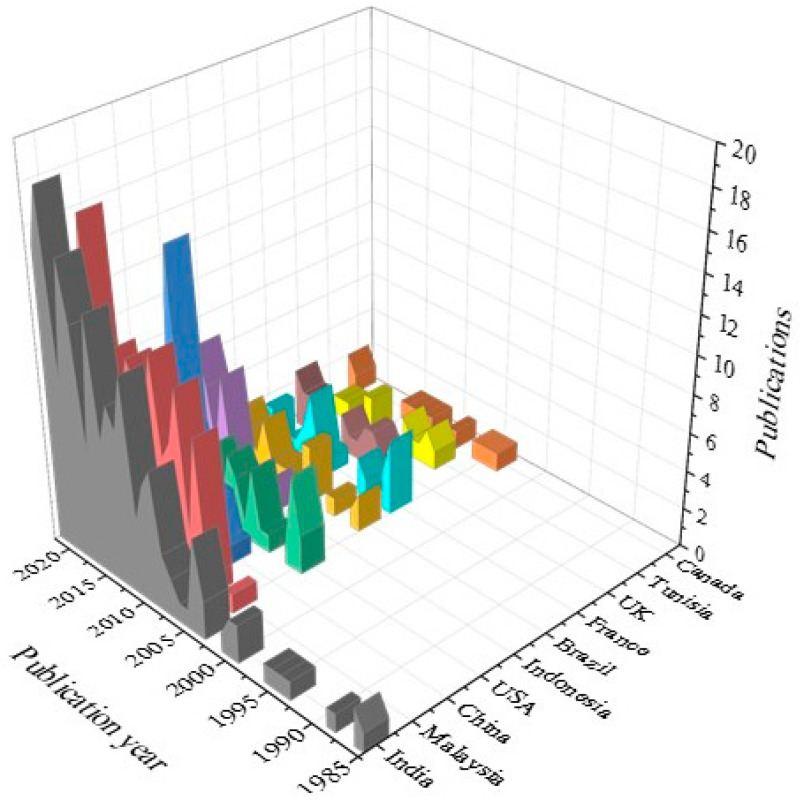
Evolution of the contribution of the top 10 publishing states. Evolution of the contribution of the top 10 publishing states between January 1982 and July 2022, elaborated based on data from VantagePoint 64-bit PRO version 14.

As shown in Figure 3, documents written by authors from all over the world were discovered in the database, totaling 62 countries, accounting for one-third of the ONU’s membership in 2021. More than 84 papers were linked to colleges in India, Malaysia, and China. (See maroon and prune colors in Figure 3). Table 2 lists the top 25 most productive states, while Figure 4 shows the top 10 publishing states since 1982. As shown in Figure 4, it is remarkable that India’s leadership in this topic began in 1985 with the works of Varma et al. (1985) [29] and continued until July 2022. Some surprising results observed were the participation of authors from lower-income states, such as Ethiopia, which ranked 156th in 2021 for per capita income per thousand USD, and, as shown in Table 2, ranked 60th in the gross domestic product (GDP). Notably, almost 50% of the authors were associated with states from the Group of Twenty. There is a considerable amount of contributions from authors from less-developed economies such as Algeria, Bangladesh, Nigeria, Iraq, Egypt, and Ethiopia. This implies that this topic offers possibilities for research worldwide regardless of the economic strength of the state.

Table 2 shows the evaluation of the publication per million population of the top 25 contributing states, and the publications per capita income was calculated using Equations (1) and (2), respectively. The results showed that Brazil, USA, and Indonesia were the three states with the highest scores with the most efficient relation of the publication per million population, whereas China displayed the lowest value, followed by Bangladesh, Nigeria, Japan, and Egypt. In the case of India, it was more efficient than states with comparatively higher incomes, such as China, Japan, and Italy, despite its larger population size.

**Figure 5:**
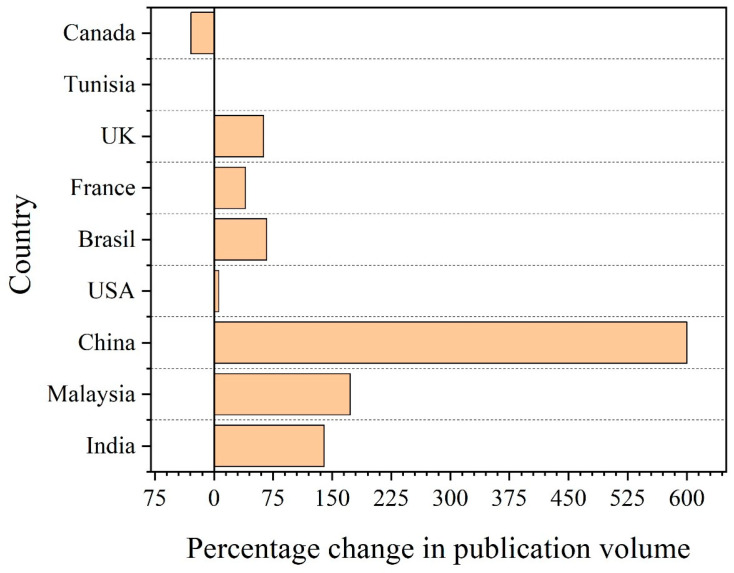
Change in publications in 2011–2021 (Equation (3)), elaborated based on data obtained using VantagePoint 64-bit PRO version 14.

Australia and Sweden had the lowest scores of the publication’s per capita income (thousand USD) with 0.01, followed by Japan and Italy, with scores of 0.02. Surprisingly, India had a score of 7.38, which is significantly high considering that the per capita income per thousand USD of Australia is around 263 times that of India. An amazing result was observed for Ethiopia, with its presence in the top 25, despite having the lowest per capita income per thousand USD in this list. Figure 5 shows the evolution of publications for the top 10 states between 2011 and 2021 calculated using Equation (3). Indonesia was excluded in the figure because all published papers for this state were published during this period. The results showed that China was the most productive nation when it came to publications, following by Malaysia, India, Brazil, and the UK.

**Figure 6:**
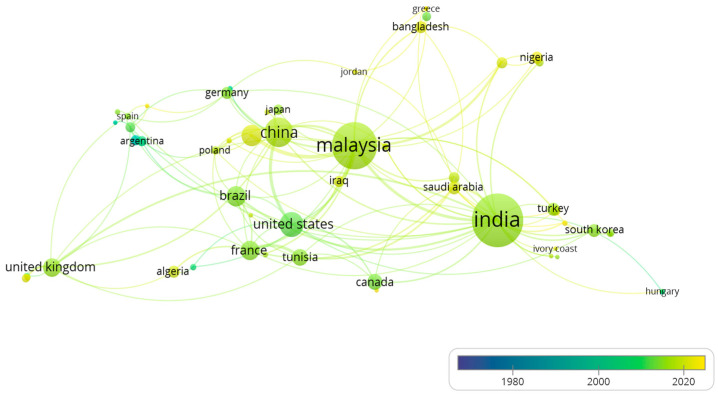
Co-authorship network of authors by state through time, elaborated by using VOSviewer version 1.6.18.

Figure 6 shows the trend of collaborations between authors from different states over time, and 59 collaboration nodes were identified. By analyzing the distance between the relevant nodes, nodes with large sizes, the distance between them, and the thickness of lines, a strong relationship was observed since 2010 between authors from Malaysia with Chinese, Indian, German, and American institutions. Since 2020, collaborations between authors from Malaysia and their colleagues from Indonesia, Bangladesh, Nigeria, and Australia have also increased. This can be attributed to the cultural affinities of Malaysian researchers with those from Indonesian or Bangladeshi and the collaborative international agreements between these states.

### 3.2. Leading Institutions

**Figure 7:**
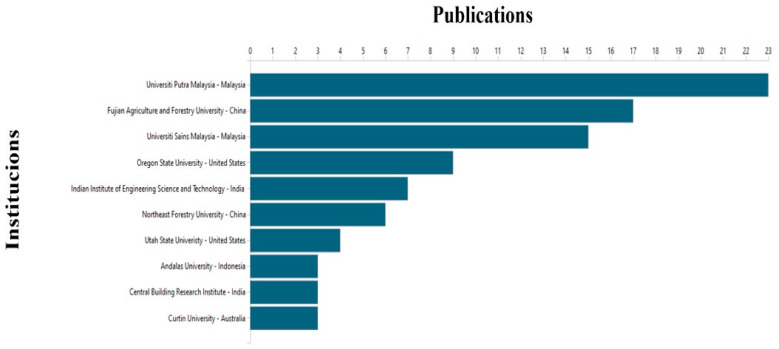
Top 10 institutions based on productivity/papers published, elaborated based on data from VantagePoint 64-bit PRO version 14.

The total of 1216 departments and institutions have previously published papers on UPR composites. As shown in Figure 7, the top 10 publishing entities were located in the leading states mentioned above, in Figure 3 and Figure 4. Remarkably, two Malaysian institutions were ranked in the top 10: Universiti Putra and Universiti Sains Malaysia, with 23 and 15 articles, respectively. These institutions were followed by two Chinese universities, Northeast Forestry University and Fujian Agriculture and Forestry University, with 6 works each of they, two American universities, Oregon State University and Utah State University with 9 and 4 articles, respectively. Further, the Indian Institute of Engineering Science and Technology and the Central Building Research Institute from India published 7 and 3 articles, respectively. The remaining institutions in the top 10 are Andalas University of Indonesia and Curtin University of Australia, with 3 publications each.

Notably, interdisciplinary work was conducted at institutions such as Universiti Putra, where research on UPR biocomposites was conducted in different divisions, such as the Department of Aerospace Engineering, the Department of Mechanical and Manufacturing Engineering, Department of Biological and Agricultural Engineering, Department of the Faculty of Engineering, and the Institute of Advanced Technology, indicating the versatility of natural fibers.

### 3.3. Leading Authors

**Figure 8:**
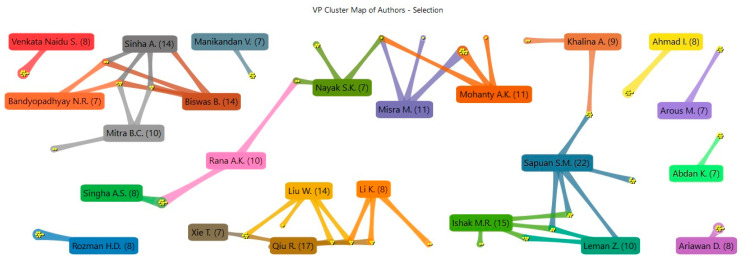
Relationship between authors that published at least seven articles, elaborated using VantagePoint 64-bit PRO version 14.

In this study, 1393 authors were identified. As shown in Figure 8, 24 authors had written at least seven papers. From these results, it is possible to observe the co-operation and co-authorship between them, which are represented by the colored lines and small balls at the end of each line, respectively. The balls represent the number of publications between the connected authors. Generally, most of these authors worked with close partners of the same institution, as with the case of the most active author, Dr. S. M. Sapuan of Universiti Putra, who published 22 documents, 8 of them in collaboration with Dr. M. R. Ishak, 7 with Dr. A. Khalina (also identified as K. Abdan), and 5 with Dr. Z. Leman, all of whom were from the same institution. Dr. R. Qiu of Fujian is the second-highest contributing author, with 17 documents. He collaborated extensively with Agriculture and Forestry University co-authors such as Dr. W. Liu on 14 texts, Dr. T. Xie on 7 texts, and Dr. K. Li, who is affiliated with the Oregon State University, on 8 documents.

Notably, there was only one cluster constituted by Dr. B. Biswas of the Indian Institute of Technology, who published 14 papers. Notably, Dr. B. Biswas has a national network with Dr. A. Sinha of Kazi Nazrul University, who was a co-author on all documents. This information suggests that, even though collaboration between authors are popular, more can be done to promote international collaboration between authors.

### 3.4. Article Citations

There was a total of 19,206 citations identified by Scopus as of July 2022. The high number of citations suggests connections by a global network of researchers interested in UPR biocomposites. Seventy documents (13.2%) have no citations at the end of July 2022. This can be attributed to 84% of these articles being published between January 2019 and July 2022, with 43% of them being published until July 2022. Table 3 summarizes the top 10 the most-cited papers, which correspond to 84.6% of citations. Two of the top 10 were reviews, including the most-cited paper, with 2463 citations, which was 12.8% of the total citations. This work, titled “Biofibres, biodegradable polymers and biocomposites: An overview,” was published in 2000 [5]. It contains detailed information about diverse types of natural fibers, including the chemical composition, mechanical properties, and a comparative analysis of the behavior of manmade fibers such as glass or carbon fibers. This work also provides detailed information about composites based on biodegradable matrices. The second review, titled “Recent developments on nanocellulose reinforced polymer nanocomposites: A review,” published in 2007 [30] focuses on useful processing techniques that develop cellulose-reinforced composites from the micro to nano scale.

Five of the top 10 publications [31,32,33,34,35] are concerned with the alkaline treatment of natural fibers, while the remaining research papers are concerned with the effects of the fiber on the mechanical or physical behaviors of UPR biocomposites. This outcome implies that the authors’ primary concerns were linked to the evaluation of UPR biocomposite behavior.

### 3.5. Source of Publications

**Figure 9:**
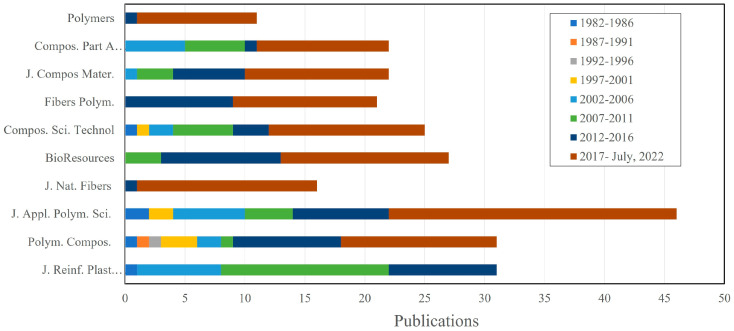
Top 10 most productive journals, elaborated based on data from VantagePoint 64-bit PRO version 14.

The analyzed papers were published across 198 journals. The journal’s subject areas included materials science, chemistry, engineering, chemistry engineering, energy, physics and astronomy, and environmental science. In the specific case of materials science, ceramics and composites were the focus. As shown in Figure 9, the Journal of Applied Polymer Science (J. Appl. Polym. Sci.) was the most productive, publishing 31 papers, followed by the Journal of Reinforced Plastics and Composites (J. Reinf. Plast. Compos.) and Polymer Composites (Polym. Compos.), who both published 31 papers each. Evidently, papers on UPR biocomposites have increased in frequency over the last 15 years. For the remaining journals, the number of papers published increased between January 2017 and July 2022. Notably, only two open access journals were included in the top 10: Polymers and BioResources. Meanwhile, only one journal was free access out of the remaining five journals, which may have affected the number of citations.

As shown in Table 4, the journal with the most-cited papers is Compos. Sci. Technol., with 2640 citations. This was followed by the Macromol. Mater. Eng. with 2480 citations. As observed in Table 3, the most-cited work was published by this journal. The next most-cited journals were J. Reinf. Plast. Compos. with 1295; J. Appl. Polym. Sci. with 1170; Compos. Part A Appl. Sci. Manuf. with 1072; Polym. Compos. with 1050; Compos. B. Eng. with 582; Mater. Des. with 558; J. Compos. Mater. with 469; and Mater. Sci. Eng. with 438 citations. Interestingly, 70% of all published papers were cited in papers ranked/listed in the top 10. This trend may be attributed to the scope of these journals, which focuses on specific areas of composites. In addition, Compos. Sci. Technol., Compos. Part A Appl. Sci. Manuf., and Compos. B. Eng. were ranked in the top 10 journals of Citescore of the subject area of materials science in the topic of ceramics and composites and had the highest values (Table 4).

Notably, there were three journals that did not make it into the top 10, despite having published more papers. In the case of J. Nat. Fibers, 86.7% of its 113 papers were cited, whereas for BioResources, all of its 244 papers were cited. Further, 80% of 101 papers published by Polymers have been cited.

### 3.6. Authors’ Keywords

**Figure 10:**
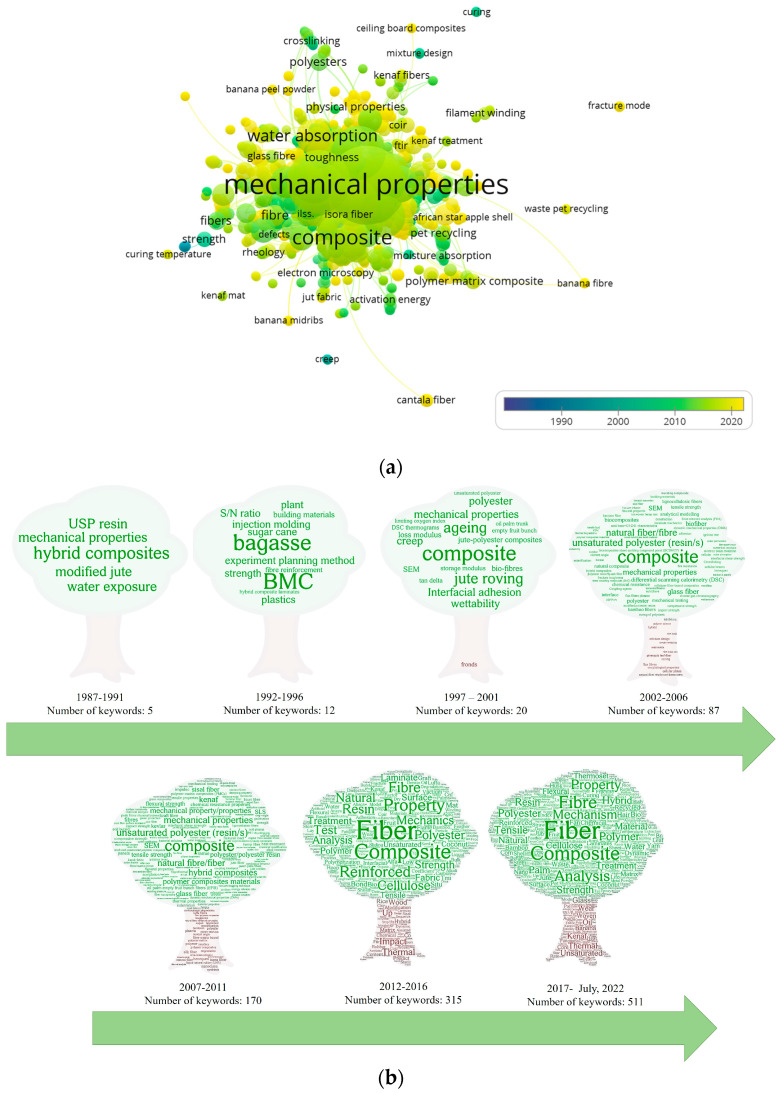
Popular keywords used by authors: (**a**) total amount of keywords used by authors, elaborated using VOSviewer version 1.6.18; and (**b**) keywords by period of time.

There were 1050 keywords identified across all analyzed articles. As observed in Figure 10a, the general cloud map of keywords over time was dominated by mechanical properties and composites. This confirms that some of the most-cited papers focused on composite evaluation. Meanwhile, Figure 10a,b showed that the variety of concepts has increased in the last 15 years, particularly in between 2017 and July 2022, in which 511 keywords were reported. The diversity of concepts can be attributed to grammatical differences, such as the use of different written forms such as fiber or fibre and alkaline or alkali treatment, the use of plural or singular words such as mechanical properties or mechanical property; or the use of the abbreviations against the complete name, particularly when mentioning a specific technique, such as scanning electron microscopy or SEM.

Even though the first recorded paper was published in 1982, the use of keywords was only registered for the first time in 1987. As observed in Figure 10b, the number of keywords used by the authors increased from 1987 to 1991. These keywords include terms such as: natural fiber sources, fiber modification, reinforcement configuration, and use of synthetic fibers to develop hybrid composites. An additional aspect identified in Figure 10b (1987–1991) is the early fabrication of hybrid composites reinforced using natural and synthetic fibers.

**Figure 11:**
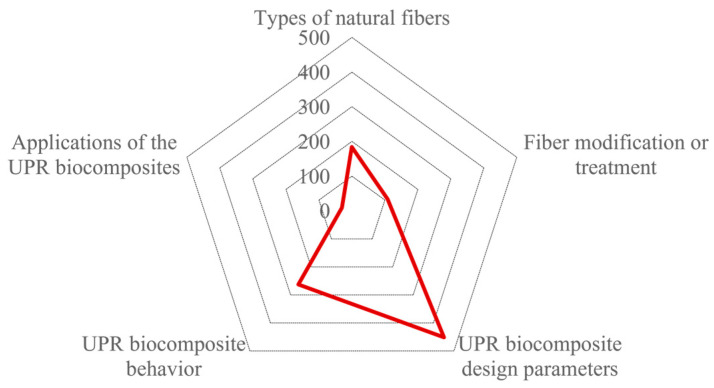
Identification of the main research areas according to authors’ keywords.

**Figure 12:**
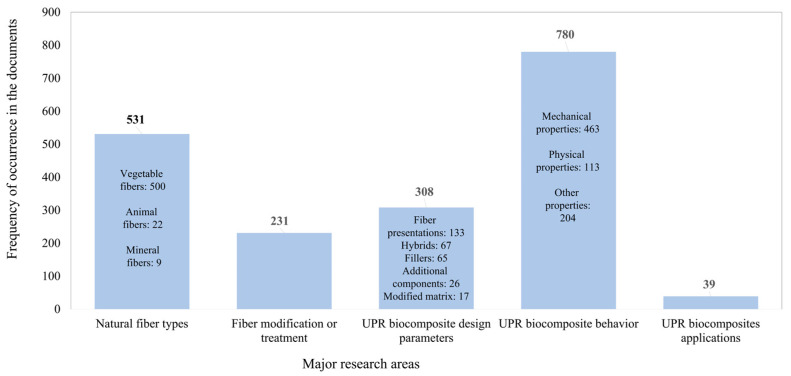
Frequency of the occurrence of main research areas in the eligible documents.

Keywords associated with composite-processing techniques such as injection molding and BMC (bulk molding compound) started appearing in the second period (1992–1996). Meanwhile, the use of specific concepts associated with composite behavior such as creep, dynamical mechanical analysis (DMA), or scanning electron microscopy (SEM) were observed in the third period (1997–2001). In addition, a notable presence corresponds to the fiber configuration, as in the case of jute roving or the use of the aspect related to interfacial adhesion, the key factor in the development of this type of biocomposite. Further, there was a considerable increase in concepts associated with composite testing, multiple origins of natural fibers, interfacial adhesion, fiber treatment, and hybrid composites in the fourth period (2002–2006). This trend was more evident in the three most recent periods (2007–2011, 2012–2016, and 2017–July 2022). Taking this evolution into consideration and based on the frequency, the authors’ keywords can be categorized into five major research areas, as illustrated in Figure 11: (1) natural fiber types, (2) fiber modification or treatment, (3) UPR biocomposite design parameters, (4) UPR biocomposite behavior, and (5) UPR biocomposite applications. Following this categorization, Figure 12 shows the frequency with which these areas appear in the eligible documents. The main research topics are, as expected, related to the evaluation of the behavior of UPR biocomposites. The area of UPR biocomposite design parameters deals with the addition of fillers, additional components, the use of the UPR matrix modified with bio-based components, the development of hybrid composites, and the use of different fiber presentations.

### 3.7. Research Areas

This section discusses the five main research areas that were previously identified based on the authors’ keyword categorization.

#### 3.7.1. Types of Natural Fibers

**Figure 13:**
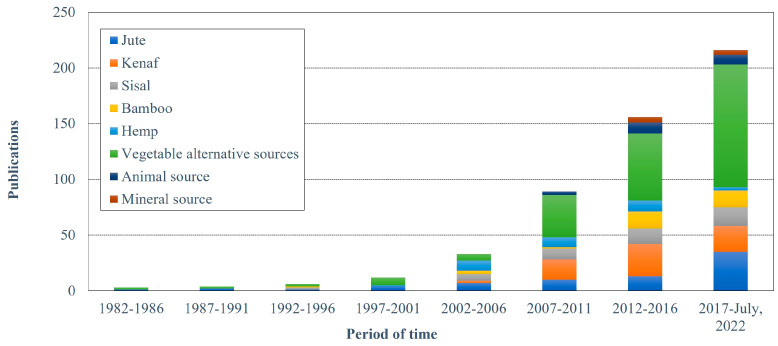
Natural fibers used between 1997 and July 2022, elaborated based on data from VantagePoint 64-bit PRO version 14.

Figure 13 displays the three main types of natural fibers discussed in the papers, according to the textile industry classification [25] and fiber science classification [14,36]: vegetable fibers, animal fibers, and mineral fibers. The case of mineral fibers under consideration in this study pertains solely to natural fibers found naturally in specific rocks. [25,36,37,38]. This research identified 129 varieties of natural fibers. In addition, the five most frequently used natural fibers were vegetable fibers such as jute, kenaf, sisal, bamboo, and hemp. These are vegetable fibers that are abundant in the international market, and have been researched by the top 10 most productive authors (Figure 8). Further, less-common vegetable fibers that include vegetable fibers commercialized in the local or national markets or isolated from wastes were considered as sources of natural reinforcement in 227 (42.7%) of the analyzed documents. However, these fibers are part of a broader variety, because this category corresponds to 76% of the identified natural fibers.

The use of fibers obtained from less-common vegetable sources was first observed in the work of Owolabi et al. (1985) [39], who evaluated short coconut fibers as reinforcements. As observed in Figure 13, this trend continued to increase over time, particularly in the two most recent periods analyzed in this work (2012–2016 and 2107–July 2022). This can be attributed to the increasing demand for reusable agro-industrial residues that traditionally did not have any additional or high-level technological applications and UPR biocomposites that offer alternative uses. These alternatives have motivated authors, and this is one of the reasons for the popularity of this topic worldwide. This has also helped identify a large variety of agro-sources since the use of coconut fibers isolated from the residue of cultivars from India [40], Malaysian bagasse residues produced during the sugar cane juice extraction [35], Nigerian African star apple (*Chrysophyllum albidum*) residues [41], or Indi.an *Cymbopogon flexuosus* residues after oil extraction [42]. These strategies support the reduction of organic charge derived from the final disposition of agro-waste [43] or food waste and promote the implementation of green and circular economy strategies with a positive economic impact on the income of farmers.

UPR biocomposites are also an opportunity to find additional alternatives to fibers that are more common in the local market, but with less impact globally, such as the Tunisian alfa fiber isolated by the esparto grass [44,45,46], called the *Stipa tenacissima* plant, Brazilian native curaua [47], and Colombian fique fibers [48].

Figure 13 shows the use of fibers isolated from animal sources, which has been observed since 2007. Notably, the use of agro-industrial residues, such as chicken feathers [49] and bovine hairs [50], has increased significantly in between the last two periods (2012–July 2022). Once again, these studies focused on reducing the environmental impact of the abundant residues by reusing them in other sectors.

With respect to mineral fibers, this study identified a heavy research focus on basalt fibers. According to Figure 13, the use of basalt fibers has increased during the last period. These fibers are formed after the rapid cooling of volcanic magma. The increasing interest in basalt fibers can be attributed to its advantages, such as UPR reinforcing owing to its mechanical, thermal, and physical behaviors [51], especially at approximately 1400 °C. In addition, basalt fibers are non-toxic and can be combined with glass fibers to produce hybrid composites [51].

#### 3.7.2. Fiber Modifications or Fiber Treatments

One of the main challenges when producing UPR biocomposites is improving the natural fiber adhesion to the UPR matrix. These difficulties can be attributed to the polar character of the fibers owing to the significant presence of hydrophilic groups in cellulosic and non-cellulosic structures with reduced wettability with the less-polar UPR matrix [10,14,48]. A common strategy to reduce the polar character of natural fibers to improve resin wettability is via fiber modification. Several studies, including complete and extensive reviews, have focused on this topic [3,5,10,14]. Generally, fiber modification, also known as fiber treatment, involves the reduction of non-cellulosic components using alkaline treatments, and the introduction of new and less-polar functional groups by reducing the OH groups of the fiber or altering the fiber surface roughness. One of the most-documented fiber modifications methods is alkaline treatment, which was used in five of the top 10 most-cited articles. There were 137 identified documents (25.8% of the total) where it was implemented. Its popularity can be attributed to the low-cost infrastructure required, ease of modification (such as by changing the alkali solution concentration, temperature, or treatment time), and ease of accessibility of the chemical products required. In some occasions, alkaline treatments were combined with other fiber modifications such as silane treatment [52] to improve the mechanical behavior of the UPR biocomposites. However, despite the popularity of alkaline treatment, 102 treatments were identified in this study.

The rest of the identified treatments can be categorized into five strategies: (a) treatment using silane agents that contain groups with a chemical affinity to UPR matrices, such as aminopropyltriethoxysilane [53,54] or 3-methacryloxypropyltrimethoxysilane [48]; (b) the use of diverse acids, anhydrides, or esters to convert OH groups by esterification or transesterification reactions into less-polar ester groups—one of the most common methods include introducing double bonds onto the natural fiber surface, which improves the resin wettability using maleic anhydride [55,56]; (c) physical processes involving the use of plasma treatment [57,58], as in the case of Sarikanat et al. (2016) [59], who used Ar and air atmospheric-pressure plasma treatments to improve the mechanical properties of flax biocomposites; (d) thermal treatment, such as steam explosions combining temperature and pressure, which reduces non-cellulosic components affecting the chemical and morphological structure of the remaining fiber structures—this process usually involves the development of binderless boards [3], but it is also useful for UPR biocomposites, as shown in the work of Brugnago et al. (2011) [60], to reduce the content of the non-cellulosic components of the vegetable fibers; and (e) interactions between natural fibers and monomers or polymers. Monomers can be useful for inducing grafting modifications onto fiber surfaces [61]. Meanwhile, polymers can be used as fiber coatings or compatibilizers. For example, the starch coating of jute fibers improves the interface contact and enhances the mechanical behavior of the composites [62]. These alternative methods create innovative uses of fiber treatment using biodegradable products with a potential positive impact on the final degradation of the material.

#### 3.7.3. UPR Biocomposite Design Parameters

There are two essential components to biocomposite design: natural reinforcement and the matrix. The main aspects of natural reinforcements are the source, reinforcement configuration, content, and use of the fiber treatment to improve the addition to the UPR matrix.

The essential parameters of the UPR matrix are the type and amount of curing agent used, the use or absence of cobalt accelerators such as the cobalt salts, the curing condition including cycles of temperature and pressure, and the use or absence of the post-curing step. This helped identify innovative alternatives that improved the mechanical behavior of the composites, utilizing strategies that include the application of a post-conforming process, such as the use of ionizing γ-irradiation on UPR composites, which enhanced the fiber/matrix interfaces [63,64] or enhanced composite behavior by the use of ultraviolet radiation for curing [65].

Notably, there has been an increasing number of studies related to the incorporation of bio-based components to modify the UPR matrix. This trend has been identified since 2005 across 17 papers (3.2%), whereas 10 of them have been published in the last analyzed period (2017–July 2022). These documents include different alternatives for reducing the environmental impacts of the matrix. In some cases, green monomers are used during UPR polymerization. A recent example is the addition of itaconic acid, ethylene glycol, and oxalic acid, which improves the mechanical and thermal properties, such as the glass transition temperature of the neat matrix [66]. On other occasions, this strategy used monomers obtained from recycled polyethylene terephthalate (PET) instead, which promotes the reuse of polymer waste [67]. A third method involves creating a matrix formed by mixing UPR and epoxidized vegetable oil, such as epoxidized soybean oil acrylate [68] or glycol-based block copolymers [63]. These alternatives could improve the biodegradability of the final material and the interface fiber/matrix modifying the mechanical behavior [63]. Another recently documented alternative is the incorporation of natural latex, such as epoxidized natural rubber [30] or Euphorbia coagulum which improved the mechanical properties and biodegradability [69].

When considering composite design, the reinforcement configuration is a key aspect that affects the mechanical behavior. In the analyzed articles, 13 reinforcement configurations were identified, including woven fabrics [66,70,71,72,73], non-woven fabrics [74,75,76], short fibers [49,77], powders [78,79], nanofibers [63,80], and nanocrystals [30], or nanostructuration with self-assembled block polymers [81].

The fiber configurations also provide opportunities to create hybrid composites that combine different types of natural fibers, such as jute–ramie [70], hemp–kenaf fiber [82], and the less-traditional banana–pandanus fibers [83]. In addition, it is common to combine natural and synthetic fibers, especially using traditional reinforcements, as glass fibers [40,84,85,86,87,88]. This tendency has also been observed in the work of Varma et al. (1985) [29]. As mentioned above, the author’s keyword hybrids are among the most used to date. Other synthetic fibers used to improve the mechanical and thermal properties of UPR hybrid biocomposites are carbon fibers [89] and Kevlar [90]. Notably, the evaluation of the hybrid fabrication parameters, such as the fiber disposition, type of fiber, number of layers, weaving conditions, hybrid fabrication, and the use of fiber binders, such as polymers that include PET or PE [36], are all relevant in the current research of UPR biocomposites.

Generally, the development of composites involves the addition of fillers. In this study, 38 different useful fillers were identified for improving the mechanical properties [40,91], wear behavior [92], cross-sectional shrinkage [91], fire retardancy [92], and sound absorption at higher frequencies [93]. These fillers originate from minerals such as montmorillonite-nanoclays and other nanoclays [40], calcium carbonate [72], industrial waste such as copper slag [94], metallic particles [95], and different types of ash derived from agro-industrial waste, such as eggshells [84], and cow horn ash [92]. Recently, nanoclays [40], ZnO nanorods that reduce water absorption [96], nanocellulose [97], or carbon nanotubes [93] have been used as nanofillers.

Different molding processes have been proposed by researchers to fabricate UPR biocomposites over time, but the leading authors did not use a specific technique. These processes include traditional open molding such as hand lay-up and spray-up. Hand lay-up is one of the most-documented methods, and it was mentioned in at least 68 articles. Some of the most recent are published in 2022 [59,98,99,100,101]. It has been used by many researchers in Indian, Malaysian, and Chinese laboratories. The second-most-documented technique is compression, which includes variations such as sheet molding compounds (SMC) [102,103,104,105] and bulk molding compounds [48]. Additional techniques include vacuum infusion [84,105], vacuum bagging [83,106,107], resin transfer molding [47,82], and pultrusion [108].

In the most recent analysis phase (2017-July 2022), a new study path arose related to precise dimensions or modifying the biocomposite surface. These technologies include laser beam and abrasive waterjet-cutting steps. However, concerns such as surface defects, delamination, or fiber degradation that affect UPR biocomposite properties must be investigated [51]. This new method shows promise for potential future applications because the components must be tight, using a secondary process that involves machining steps.

#### 3.7.4. UPR Biocomposite Behavior

The effects of natural fibers on the mechanical behaviors of UPR biocomposites were explored in 87.2% of the identified papers. These evaluations have been predominant since 1982 and are mainly related to flexural and tensile tests. For tensile testing, researchers have attempted to establish the effects of untreated and treated natural fibers on the composite behavior [30,41,62,63,70,71,84,89,91,103,105]. Additionally, other types of tensile testing based on the evaluation of a single fiber or few fibers embedded in the matrix are useful for evaluating the matrix wettability, fiber/matrix adhesion, and efficiency of the fiber treatment [109]. The mechanical analyses also included other tests, such as the compression test [2,108], DMA [29,60,64,74], hardness test [41,66,91,110], impact test [2,62,111], and wear test [47,84].

The second-most-documented UPR biocomposite evaluation identified in this study is the thermal behavior. The most common technique is thermogravimetric analysis (TGA), which appeared in at least 21.3% of the articles. Further, fire retardancy behavior was mentioned in 3.0% of the documents, half of which were published in the last period of the analysis (2019 and July 2022). Their central measurements corresponded to the limiting oxygen index. Kumar et al. (1997) [112] was the first to publish about the fire retardancy of oil-palm- and glass-fiber-reinforced UPR hybrid composites.

With respect to physical evaluations, the most common assays corresponded to the determination of water uptake or moisture capture. These evaluations were identified in 26.9% of the documents. These assessments are important for identifying the potential applications of UPR biocomposites and determining the effects of the fiber modification on biocomposite behaviors owing to the reduced polar behavior of the natural fibers. There were broad alternatives that included the evaluation of sample weight after immersion in fresh water [62,70,113] or seawater [43,108], or variations in the thickness of samples caused by swelling [70].

Other evaluations of UPR biocomposites include the chemical resistance to acidic or basic environments [107,114], acoustical testing [74], antibacterial activity [47], biodegradability [69,102], ballistic behavior [90], dielectric testing [49], electrical conductivity, and thermal insulation [95]. Surprisingly, there was only one paper which discussed the biodegradation or degradation of UPR composites, in this case by photodegradation [113]. This lack of research suggests the need for further work on the end-consumer cycle of UPR biocomposites.

#### 3.7.5. Applications of the UPR Biocomposites

Concerns regarding the potential applications of UPR biocomposites have been present since the first publication; the first mention identified in this study corresponds to the production of water-resistant chipboards and fiber hardboards [8]. This interest has continued to date. The bibliometric analysis identified 60 different mentions of viable applications in the analyzed documents, half of which appeared between 2017 and July 2022. The fabrication of automotive interior applications [2] was the most-documented application, appearing in 12 works, half of which were published between 2017 and July 2022. Interior construction and furniture are other important sectors in which UPR biocomposites have been considered for application [67,103,115]. For example, new products can be developed as lightweight and mechanically strong materials for earthquake-resistant wall panels [116] or marine transport applications [117], and as round rods used for insulating material applications. Information about potential applications were provided in 39 of the analyzed papers (7.3%), and 17.9% of these documents included authors affiliated with non-university institutions. This indicates the relevance of improving collaboration between research centers, industries, and universities to develop UPR composites suitable for application.

### 3.8. Opportunities for Future Research

As our work has shown, UPR biocomposites are a dynamic, global phenomenon that has offered opportunities to hundreds of researchers worldwide, particularly when using waste from food production, industrial byproducts, and post-consumer materials. There was a tendency noted by other writers when analyzing certain types of residues, such as the example of olive stones investigated by Valdez et al. [118]. The development of bio-based composites based on a circular economy and green chemistry can also be supported by the utilization of new raw materials, as recently commented by authors such as Daniel-Mkpume et al. [41] and Mujtaba et al. [119]. The potential for further research in this topic includes the following:

- Increasing the use of bio-based matrices in the production of UPR biocomposites. Some of the research cited in this study [67,68,120] have investigated this line of inquiry, but it has the potential to grow in the next years, especially given the alternative of inserting natural components during UPR polymerization. Alternatives have been studied further in various polymer matrices [14], as well as in other thermo-set matrices, such as the bio-based epoxy resin [121].
- Analyzing the environmental effect of the waste produced by fiber treatment, and evaluating less environmental detrimental processes which involve the reduction of the water and energy consumption, and the reduction or substitution of hazardous and difficult-to-dispose substances.
- Continuing the development of UPR hybrid composites based on mixtures of natural fibers and natural synthetic fibers, as well as investigating the potential to include nanofillers or nanoreinforcements.
- Using nanoreinforcements or nanofillers as a preferred reinforcement for the UPR matrix [68]. These nanoreinforcements may be adding during the manufacture of UPR biocomposite materials or during matrix polymerization, depending on their features.

Although this study indicates a growing interest in the field, further research is still required, especially given the transition from lab prototypes to semi-industrial- or industrial-scale applications. It is necessary to increase the participation of researchers from various industrial sectors to advance this topic.

Additional themes to investigate in relation to UPR biocomposites that were not identified in the examined documents but are of interest in material science research and have also been considered for other biocomposites [122] are: (1) evaluations of carbon neutrality, (2) evaluations of life cycle, (3) the analysis of circular economy, and (4) the analysis of manufacturing energy use. Future research investigations on these subjects should be considered, particularly because of the growing concerns over the role of material sciences in combating climate change.

## 4. Conclusions

This paper offered a quick and brief overview on the status of the development of UPR biocomposites using bibliometric analysis. We employed macro- and microlevel studies to discover the worldwide relevance of this topic, beginning with identifying the leading states where it is most relevant and significant, and progressing to identifying relevant research fields by analyzing the author’s keywords and selected papers. It was discovered that one-third of the United Nations member countries had been active in UPR biocomposite research. The cubic regression of the publishing tendency shows that there is an active and growing interest in this area. The most active research correlates to studies undertaken in developing countries such as India, China, and Malaysia, which imply a potential future important impact on economies due to the association between research work per capita income and GDP. The five active research areas were identified through bibliometric analysis and an in-depth evaluation of the selected paper: (1) the type of natural fiber, (2) fiber modification or treatment, (3) UPR biocomposite design parameters, (4) UPR biocomposite design parameters, and (5) applications. It was possible to identify and suggest topics for future works for each of them, which included increasing the use of bio-based matrices in the production of UPR biocomposites, evaluating the environmental impact of waste produced due to fiber treatment, and implementing treatment that considers less use of water and energy resources. Furthermore, in addition to these tactics, this work illustrates the importance of continuing working to enhance the building of stronger industrial and academic teamwork, aided by international co-operation.

## Figures and Tables

**Table 1:** Types of documents.

Documents	Amount
Article	563
Conference paper	191
Book chapter	24
Review	23
Conference review	18
Data paper	1

**Table 2:** Evaluation of the efficiency of the states.

Rank	State	Number of Publications	GDP Ranking in 2021 ^1^	Publications per Million Population (Equation (1))	Publications per Capita Income Thousand USD (Equation (2))
1	India	168	6	1.21 × 10^−4^	7.38
2	Malaysia	130	37	4.00 × 10^−3^	1.14
3	People’s R. of China	52	2	3.70 × 10^−5^	0.08
4	United States	36	1	1.08 × 10^−1^	0.05
5	Indonesia	27	16	9.77 × 10^−2^	0.63
6	Brazil	25	12	1.17 × 10^−1^	0.33
7	France	23	7	3.41 × 10^−3^	0.05
8	United Kingdom	21	5	3.12 × 10^−4^	0.03
9	Tunisia	17	88	1.42 × 10^−3^	0.43
10	Canada	14	9	3.66 × 10^−4^	0.03
11	Germany	11	4	1.32 × 10^−4^	0.02
12	Saudi Arabia	10	18	2.83 × 10^−4^	0.04
13	Korea, Rep. of	10	10	1.93 × 10^−4^	0.03
14	Turkey	10	19	1.18 × 10^−4^	0.10
15	Algeria	9	57	2.02 × 10^−4^	0.24
16	Bangladesh	9	32	5.40 × 10^−5^	0.36
17	Nigeria	9	30	4.30 × 10^−5^	0.43
18	Iraq	8	52	1.94 × 10^−4^	0.16
19	Australia	7	13	2.72 × 10^−4^	0.01
20	Egypt, Arab Rep.	7	33	6.70 × 10^−5^	0.18
21	Japan	7	3	5.60 × 10^−5^	0.02
22	Argentina	6	26	1.31 × 10^−4^	0.06
23	Ethiopia	6	60	5.10 × 10^−5^	0.64
24	Italy	6	8	1.02 × 10^−4^	0.02
25	Sweden	6	22	5.76 × 10^−4^	0.01

^1^ Information from: chrome-extension://efaidnbmnnnibpcajpcglclefindmkaj/https://databankfiles.worldbank.org/data/download/GDP.pdf (accessed on 5 December 2022).

**Table 3:** Top 10 most-cited papers.

Rank	Authors	Paper Title	Year of Publication	Journal Title (Abbreviation ^1^)	Citations ^2^
1.	Mohanty A.K.; Misra M.; Hinrichsen G.	Biofibres, biodegradable polymers and biocomposites: An overview	2000	Macromolecular Materials and Engineering (Macromol. Mater. Eng.)	2463
2	Dhakal H.N.; Zhang Z.Y.; Richardson M.O.W.	Effect of water absorption on the mechanical properties of hemp fibre reinforced unsaturated polyester composites	2007	Composites Science and Technology (Compos. Sci. Technol.)	1061
3	Kargarzadeh H.; Mariano M.; Huang J.; Lin N.; Ahmad I.; Dufresne A.; Thomas S.	Recent developments on nanocellulose reinforced polymer nanocomposites: A review	2017	Polymer (Polymer)	348
4	Vilay V.; Mariatti M.; Mat Taib R.; Todo M.	Effect of fiber surface treatment and fiber loading on the properties of bagasse fiber-reinforced unsaturated polyester composites	2008	Composites Science and Technology (Compos. Sci. Technol.)	291
5	Manfredi L.B.; Rodríguez E.S.; Wladyka-Przybylak M.; Vázquez A.	Thermal degradation and fire resistance of unsaturated polyester, modified acrylic resins and their composites with natural fibres	2006	Polymer Degradation and Stability (Polym. Degrad. Stab.)	267
6	Athijayamani A.; Thiruchitrambalam M.; Natarajan U.; Pazhanivel B.	Effect of moisture absorption on the mechanical properties of randomly oriented natural fibers/polyester hybrid composite	2009	Materials Science and Engineering A (Mater. Sci. Eng. A)	256
7	Khalil H.P.S.A.; Ismail H.; Rozman H.D.; Ahmad M.N.	Effect of acetylation on interfacial shear strength between plant fibres and various matrices	2001	European Polymer Journal (Eur. Polym. J.)	241
8	Shanmugam D.; Thiruchitrambalam M.	Static and dynamic mechanical properties of alkali treated unidirectional continuous Palmyra Palm Leaf Stalk Fiber/jute fiber reinforced hybrid polyester composites	2013	Materials and Design (Mater. Des.)	223
9	Aziz S.H.; Ansell M.P.; Clarke S.J.; Panteny S.R.	Modified polyester resins for natural fibre composites	2005	Composites Science and Technology (Compos. Sci. Technol.)	219
10	Baley C.; Busnel F.; Grohens Y.; Sire O.	Influence of chemical treatments on surface properties and adhesion of flax fibre-polyester resin	2006	Composites Part A: Applied Science and Manufacturing (Compos. Part A Appl. Sci. Manuf.)	206

^1^ Note: Journal abbreviations were made using ISO 4. ^2^ Note: Citations reported by Scopus system until July 2022.

**Table 4:** Top 10 most-cited journals, elaborated based on data from VantagePoint 64-bit PRO version 14.

Rank	Journal Title Abbreviations ^1^	Citations ^2^	Total Papers	Cited Papers	CiteScore 2021 ^3^
1	Compos. Sci. Technol.	2640	13	13	14.7
2	Macromol. Mater. Eng.	2480	3	2	6.2
3	J. Reinf. Plast. Compos.	1295	31	31	6.2
4	J. Appl. Polym. Sci.	1170	24	24	5.0
5	Compos. Part A Appl. Sci. Manuf.	1072	11	11	13.7
6	Polym. Compos.	1050	31	28	5.7
7	Compos. B. Eng.	582	8	7	18.6
8	Mater. Des.	558	7	7	13.2
9	J. Compos Mater.	469	12	12	4.7
10	Mater. Sci. Eng. A	438	3	3	9.4

^1^ Note: Journal abbreviations were made using ISO 4. ^2^ Note: Citations until 1 August 2022. ^3^ Note: Retrieved 29 November 2022, from www.scopus.com (accessed on 12 December 2022).

## Data Availability

The data reported are available by contacting the corresponding author.

## References

[1] Market Analysis Report. Unsaturated Polyester Resin Market Size, Share & Trends Analysis Report by Product (DCPD, Orthophthalic, Isophthalic), by End-Use, by Form (Liquid Form, Powder Form), by Region, and Segment Forecasts. https://www.grandviewresearch.com/industry-analysis/unsaturated-polyester-resin-upr-market.

[2] Aynalem G.F., Sirahbizu B. (2021). Effect of Al_2_O_3_ on the tensile and impact strength of flax/unsaturated polyester composite with emphasis on automobile body applications. Adv. Mater. Sci..

[3] Gallego R.Z., Vélez-Acosta L.M., Gómez-Hoyos C., Velásquez-Cock J., Serpa-Guerra A., Rojo P.G., Rajeshkumar G., Devnani G., Sinha S., Sanjay M.R., Siengchin S. (2022). Manufacturing aspects of bast fiber-based composites. Bast Fibers and Their Composites.

[4] Ali M.F., Hossain M.S., Ahmed S., Sarwaruddin Chowdhury A.M. (2021). Fabrication and characterization of eco-friendly composite materials from natural animal fibers. Heliyon.

[5] Mohanty A.K., Misra M., Hinrichsen G. (2000). Biofibres, biodegradable polymers and biocomposites: An overview. Macromol. Mater. Eng..

[6] Rodriguez L.J., Peças P., Carvalho H., Orrego C.A. (2020). A literature review on life cycle tools fostering holistic sustainability assessment: An application in biocomposite materials. J. Environ. Manag..

[7] Rafiee K., Schritt H., Pleissner D., Kaur G., Brar S.K. (2021). Biodegradable green composites: It’s never too late to mend. Curr. Opin. Green Sustain. Chem..

[8] Schaudy R., Proksch E. (1982). Wood-plastic combinations with high dimensional stability. Ind. Eng. Chem. Res..

[9] Gañán P., Marín D., Builes D., Thomas S., Chirayil C. (2023). Potential of natural fiber in unsaturated polyester biocomposite application. Applications of Unsaturated Polyester Resins.

[10] Bledzki A.K., Gassan J. (1999). Composites reinforced with cellulose based fibres. Prog. Polym. Sci..

[11] Lammel-Lindemann J., Dourado I.A., Shanklin J., Rodriguez C.A., Catalani L.H., Dean D. (2020). Photocrosslinking-based 3D printing of unsaturated polyesters from isosorbide: A new material for resorbable medical devices. Bioprinting.

[12] Kumar R., Ul Haq M.I., Raina A., Anand A. (2019). Industrial applications of natural fibre-reinforced polymer composites-challenges and opportunities. Int. J. Sustain. Eng..

[13] Xiao B., Yang Y., Wu X., Liao M., Nishida R., Hamada H. (2015). Hybrid laminated composites molded by spray lay-up process. Fibers Polym..

[14] Vinod A., Sanjay M.R., Suchart S., Jyotishkumar P. (2020). Renewable and sustainable biobased materials: An assessment on biofibers, biofilms, biopolymers and biocomposites. J. Clean. Prod..

[15] Mohammed L., Ansari M.N., Pua G., Jawaid M., Islam M.S. (2015). A review on natural fiber reinforced polymer composite and its applications. Int. J. Polym. Sci..

[16] Newman R.H., Battley M.A., Carpenter J.E., Le Guen M.J. (2012). Energy loss in a unidirectional flax-polyester composite subjected to multiple tensile load-unload cycles. J. Mater. Sci..

[17] Zhang Z., Hu G., Mu X., Li K. (2022). From low carbon to carbon neutrality: A bibliometric analysis of the status, evolution and development trend. J. Environ. Manag..

[18] Bastos de Sousa F.D. (2022). A simplified bibliometric mapping and analysis about sustainable polymers. Mater. Today Proc..

[19] Merigó J.M., Yang J.-B. (2017). A bibliometric analysis of operations research and management science. Omega.

[20] Yang H., Liu L., Yang W., Liu H., Ahmad W., Ahmad H., Aslam F., Joyklad P. (2022). A comprehensive overview of geopolymer composites: A bibliometric analysis and literature review. Case Stud. Constr. Mater..

[21] Nordin A.H., Ngadi N., Ilyas A.R., Nabgan W., Norfarhana A.S. (2023). Starch-based plastics: A bibliometric analysis. Mater. Today Proc..

[22] Aria M., Cuccurullo C. (2017). bibliometrix: An R-tool for comprehensive science mapping analysis. J. Informetr..

[23] Aghaei Chadegani A., Salehi H., Yunus M., Farhadi H., Farhadi M., Ale Ebrahim N. (2013). A comparison between two main academic literature collections: Web of science and Scopus databases. Asian Soc. Sci..

[24] Bergman E.M.L. (2012). Finding citations to social work literature: The relative benefits of using Web of Science, Scopus, or Google Scholar. J. Acad. Librariansh..

[25] Casa Aruta F. (1969). Diccionario de la Industria Textil.

[26] Cobo M.J., López-Herrera A.G., Herrera-Viedma E., Herrera F. (2012). SciMAT: A new science mapping analysis software tool. J. Am. Soc. Inf. Sci..

[27] Amin M.T., Khan F., Amyotte P. (2019). A bibliometric review of process safety and risk analysis. Process Saf. Environ. Prot..

[28] The Worldbank. Population. https://data.worldbank.org/indicator/SP.POP.TOTL?locations=LY&most_recent_value_desc=true.

[29] Varma D., Varma M., Varma I. (1985). Coir fibres II: Evaluation as a reinforcement in unsaturated polyester resin composites. J. Reinf. Plast. Compos..

[30] Kargarzadeh H., Mariano M., Huang J., Lin N., Ahmad I., Dufresne A., Thomas S. (2017). Recent developments on nanocellulose reinforced polymer nanocomposites: A review. Polymer.

[31] Shanmugam D., Thiruchitrambalam M. (2013). Static and dynamic mechanical properties of alkali treated unidirectional continuous Palmyra Palm Leaf Stalk fiber/jute fiber reinforced hybrid polyester composites. Mater. Des..

[32] Dhakal H.N., Zhang Z.Y., Richardson M.O.W. (2007). Effect of water absorption on the mechanical properties of hemp fibre reinforced unsaturated polyester composites. Compos. Sci. Technol..

[33] Baley C., Busnel F., Grohens Y., Sire O. (2006). Influence of chemical treatments on surface properties and adhesion of flax fibre–polyester resin. Compos. Part A Appl. Sci. Manuf..

[34] Aziz S.H., Ansell M.P., Clarke S., Panteny S.R. (2005). Modified polyester resins for natural fibre composites. Compos. Sci. Technol..

[35] Vilay V., Mariatti M., Mat Taib R., Todo M. (2008). Effect of fiber surface treatment and fiber loading on the properties of bagasse fiber–reinforced unsaturated polyester composites. Compos. Sci. Technol..

[36] Müssig J., Slootmaker T., Müssing J. (2010). Types of fibre. Book Industrial Applications of Natural Fibres. Structure, Properties and Technical Applications.

[37] International Mineralogical Association and the French Agency for Environmental and Occupational Health Safety (AFSSET) Safety in the use of mineral and synthetic fibres. Proceedings of the Working Document and Report of the Meeting of Experts on Safety in the Use of Mineral and Synthetic Fibres.

[38] Whittaker E.J.W., Eichnorn S.J., Hearle J.W.S., Jaffe M., Kikutani T. (2009). Structure and properties of asbestos. Handbook of Textile Fibre Structure. Volume 2: Natural, Regenerated, Inorganic and Specialist Fibres.

[39] Owolabi O., Czvikovszky T., Kovács I. (1985). Coconut-fiber-reinforced thermosetting plastics. J. Appl. Polym. Sci..

[40] Rajini N., Jappes J.W., Rajakarunakaran S., Jeyaraj P. (2012). Mechanical and free vibration properties of montmorillonite clay dispersed with naturally woven coconut sheath composite. J. Reinf. Plast. Compos..

[41] Daniel-Mkpume C.C., Ahaiwe R.C., Ifenatuorah C.L., Ezema Ike-Ezeet I.C., Sunday Aigbodion V., Egoigwe S.V., Okonkw E.G. (2022). Potential end of life application of African star apple shell and waste toner powder as composite filler materials. J. Mater. Cycles Waste Manag..

[42] Sundaram R.S., Rajamoni R., Suyambulingam I., Isaac R. (2022). Comprehensive characterization of industrially discarded cymbopogon flexuosus stem fiber reinforced unsaturated polyester composites: Effect of fiber length and weight fraction. J. Nat. Fibers.

[43] Mansingh B.B., Binoj J.S., Anbazhagan V.N., Hassan S.A., Goh K.L., Siengchin S., Sanjay M.R., Jaafar M.M., Liu Y. (2022). Characterization of Cocos nucifera L. peduncle fiber reinforced polymer composites for lightweight sustainable applications. J. Appl. Polym. Sci..

[44] Triki A., Guicha M., Ben Hassen M., Arous M. (2013). Comparative study of the dielectric properties of natural-fiber-matrix composites and E-glass-matrix composites. J. Appl. Polym. Sci..

[45] Triki A., Dittmer J., Hassen M., Arous M., Bulou A., Gargouri M. (2016). Spectroscopy analyses of hybrid unsaturated polyester composite reinforced by Alfa, wool, and thermo-binder fibres. Polym. Sci. Series A.

[46] Ellouze D.A., Jesson M.-L., Abel R., Ben Cheikh R., Watts J.F. (2020). An advance in the use of natural resources: Characterisation of the quality of impregnation of bleached alfa pulpboard by unsaturated polyester resin and evaluation of the obtained composite material’s properties. Ind. Crops Prod..

[47] Premkumar T., Irulappasamy S., Neis P., Amico S.C., Ferreira N.F., Winowlin Jappes J.T. (2019). Experimental design and theoretical analysis on the various tribological responses of curauá/polyester composites. Mater. Res. Express.

[48] Gañán P., Mondragon I. (2004). Fique fiber-reinforced polyester composites: Effects of fiber surface treatments on mechanical behavior. J. Mater. Sci..

[49] Hamdan S., Kiew K.S., Rahman M.R. (2014). Dielectric properties of maleic anhydride modified unsaturated polyester composites reinforced with chicken feather fibre. Int. J. Interact. Des. Manuf..

[50] Agbeboh N.I., OlaJide J.L., Oladele I.O., Babarinsa S.O. (2019). Kinetics of moisture sorption and improved tribological performance of keratinous fiber-reinforced ortho-phthalic polyester biocomposites. J. Nat. Fibers.

[51] Masoud F., Sapuan S.M., Ariffin M.K.A.M., Nukman Y., Bayraktar E. (2021). Experimental analysis of kerf taper angle in cutting process of sugar palm fiber reinforced unsaturated polyester composites with laser beam and abrasive water jet cutting technologies. Polymers.

[52] Mohd Ghaztar M.M., Nik Ibrahim N.N.I., Romli A.Z. (2022). Sodium hydroxide/silane treated kenaf fibre in unsaturated polyester matrix: Effects of fibres length and fibres loading towards. The composites flexural and morphological properties. J. Mech. Eng..

[53] Rana A.K., Singh A.S. (2022). Development and evaluation of physico-chemical properties of functionalized Cannabis indica fibers reinforced bio-polymer composites. J. Nat. Fibers.

[54] Rajkumar S., Tjong J., Nayak S.K., Sain M. (2015). Wetting behavior of soy-based resin and unsaturated polyester on surface-modified sisal fiber mat. J. Reinf. Plast. Compos..

[55] Bessadok A., Roudesli S., Marais S., Follain N., Lebrun L. (2009). Alfa fibres for unsaturated polyester composites reinforcement: Effects of chemical treatments on mechanical and permeation properties. Compos. Part A Appl. Sci. Manuf..

[56] Rozman H.D., Musa L., Abubakar A. (2005). Rice husk-polyester composites: The effect of chemical modification of rice husk on the mechanical and dimensional stability properties. J. Appl. Polym. Sci..

[57] Bozaci E., Sever K., Sarikanat M., Seki Y., Demir A., Ozdogan E., Tavman I. (2013). Effects of the atmospheric plasma treatments on surface and mechanical properties of flax fiber and adhesion between fiber-matrix for composite materials. Compos. Part B Eng..

[58] Sinha E., Panigrahi S. (2009). Effect of plasma treatment on structure, wettability of jute fiber and flexural strength of its composite. J. Compos. Mater..

[59] Sarikanat M., Seki Y., Sever K., Bozaci E., Demir A., Ozdogan E. (2016). The effect of argon and air plasma treatment of flax fiber on mechanical properties of reinforced polyester composite. J. Ind. Text..

[60] Brugnago R.J., Satyanarayana K.G., Wypych F., Pereira Ramos L. (2011). The effect of steam explosion on the production of sugarcane bagasse/polyester composites. Compos. Part A Appl. Sci. Manuf..

[61] Fei M.E., Xie T., Liu W., Chen H., Qiu R. (2017). Surface grafting of bamboo fibers with 1,2-epoxy-4-vinylcyclohexane for reinforcing unsaturated polyester. Cellulose.

[62] Swain P., Rahul P., Chouhan D., Mohanty S.P. (2022). Effect of fibre surface treatment on the mechanical properties of jute fibre reinforced unsaturated polyester omposite. J. Nat. Fibers.

[63] Builes D.H., Labidi J., Eceiza A., Mondragon I., Tercjak A. (2013). Unsaturated polyester nanocomposites modified with fibrillated cellulose and PEO-b-PPO-b-PEO block copolymer. Compos. Sci. Technol..

[64] Dash B., Rana A., Mishra H.K., Nayak S.K., Mishra S.C., Tripathyet S.S. (1999). Novel, low-cost jute-polyester composites. Part 1: Processing, mechanical properties, and SEM analysis. Polym. Compos..

[65] Hussein M.A., Tay G.S., Rozman H.D. (2012). Photo-fabricated unsaturated polyester resin composites reinforced by kenaf fibers, synthesis and characterization. J. Appl. Polym. Sci..

[66] Li Y., Qu J., Dai Z., Jiang J., Fu J., Fu F., Liu X. (2022). Highly bio-based unsaturated polyester resins with improved performance by incorporating isosorbide into the polyester prepolymer. Macromol. Mater. Eng..

[67] Wilson N., Almaral J., Ortiz R., Hurtado Macía A., Flores Ramírez N., Aguilar Palazuelos E., Flores Valenzuela J., Castro Beltrán A., Alvarado Beltrán C.G. (2021). Physical and mechanical properties of unsaturated polyester resin matrix from recycled PET (based PG) with corn straw fiber. J. Appl. Polym. Sci..

[68] Mohanty P., Behera D., Panda S.K., Kumar Bastia T., Rath P. (2020). Hybrid composite laminates from UPE/ESO. A blend reinforced with chitosan and bamboo fiber: A study of mechanical and thermal properties. Asian J. Chem..

[69] Kumari S., Kumar R., Rai B., Sirohi S., Shukla L., Kumar G. (2021). Development and study of biodegradability of Euphorbia Coagulum modified polyester composite reinforced with bamboo fiber. Fibers Polym..

[70] Hadi A.E., Hamdan M.H.M., Siregar J.P., Junid R., Tezara C., Purna Irawan A., Fitriyana D.F., Rihayat T. (2021). Application of micromechanical modelling for the evaluation of elastic moduli of hybrid woven jute-ramie reinforced unsaturated polyester composites. Polymers.

[71] Dress G.A., Woldemariam M.H., Redda D.T. (2021). Influence of fiber orientation on impact resistance behavior of woven sisal fiber reinforced polyester composite. Adv. Mater. Sci. Eng..

[72] Arumugam C., Arumugam S., Muthusamy S. (2020). Mechanical, thermal and morphological properties of unsaturated polyester/chemically treated woven kenaf fiber/AgNPs@PVA hybrid nanobiocomposites for automotive applications. J. Mater. Res. Technol..

[73] Nurazzi N.M., Khalina A., Sapuan S.M., Ilyas R.A., Rafiqah S.A., Hanafee Z.M. (2020). Thermal properties of treated sugar palm yarn/glass fiber reinforced unsaturated polyester hybrid composites. J. Mater. Res. Technol..

[74] Miah M.S., Yu J., Yang Y., Memon H., Rashid M.A. (2021). Durability and notch sensitivity analysis of environmental ageing induced glass fibre mat and kenaf fibre mat-reinforced composites. J. Ind. Text..

[75] Kenned J.J., Sankaranarayanasamy K., Kalyanavalli V., Suresh Kumar C. (2020). Characterization of indentation damage resistance and thermal diffusivity of needle-punched Musa sapientum cellulosic fiber/unsaturated polyester composite laminates using IR thermography. Polym. Compos..

[76] Kakati N., Assanvo E.F., Kalita D. (2019). Synthesis and performance evaluation of unsaturated polyester blends of resins and its application on non-woven/fabric jute fibers reinforced composites. J. Polym. Environ..

[77] Sawpan M.A., Pickering K.L., Fernyhough A. (2012). Flexural properties of hemp fibre reinforced polylactide and unsaturated polyester composites. Compos. Part A Appl. Sci. Manuf..

[78] Sari N.H., Suteja S., Fudholi A., Zamzuriadi A., Sulistyowati E.D., Pandiatmi P., Sinarep S., Zainuri A. (2021). Morphology and mechanical properties of coconut shell powder-filled untreated cornhusk fibre-unsaturated polyester composites. Polymer.

[79] Rahman M.R., Hamdan S., Ngaini Z.B., Jayamani E., Kakar A., Bakri M.K.B., Yusof F.A.B.M. (2019). Cellulose fiber-reinforced thermosetting composites: Impact of cyanoethyl modification on mechanical, thermal and morphological properties. Polym. Bull..

[80] Grzetic J., Rančić M., Pavlovic V., Rakić V.M., Stevanović S., Djonlagić J., Marinković A.D. (2018). Cross-linkable modified nanocellulose/polyester resin-based composites: Effect of unsaturated fatty acid nanocellulose modification on material performances. Macromol. Mater. Eng..

[81] Builes D.H., Hernández-Ortiz J.P., Corcuera M.A., Mondragon I., Tercjak A. (2014). Effect of poly(ethylene oxide) homopolymer and two different poly(ethylene oxide-b-poly(propylene oxide)-b-poly(ethylene oxide) triblock copolymers on morphological, optical, and mechanical properties of nanostructured unsaturated polyester. ACS Appl. Mater. Interfaces.

[82] Rouison D., Sain M., Couturier M. (2004). Resin transfer molding of natural fiber reinforced composites: Cure simulation. Compos. Sci. Technol..

[83] Mariatti M., Jannah M., Abu Bakar A., Abdul Khalil H.P.S. (2008). Properties of banana and pandanus woven fabric reinforced unsaturated polyester composites. J. Compos. Mater..

[84] Kumar N., Walia R., Angra S. (2022). An interactive study on wear behaviour and mechanical properties of carbonized eggshells filler loaded glass-jute reinforced polyester hybrid bio-composites. Int. J. Interact. Des. Manuf..

[85] Panigrahi S., Oraji R., Arachilage K., Kushwaha R.L., Panigrahy B.S. (2009). Characteristics of hybrid fibre-composites boards for potential structural application. SAE Int. J. Commer. Veh..

[86] Sapuan S.M., Aulia H.S., Ilyas R.A., Atiqah A., Dele-Afolabi T.T., Nurazzi M.N., Supian A.B.M., Atikah M.S.N. (2020). Mechanical properties of longitudinal basalt/woven-glass-fiber-reinforced unsaturated polyester-resin hybrid composites. Polymers.

[87] Ali A., Quan W.N., Arifin F., Rassiah K., Othman F., Hazin S., Megat Ahmad M.H. (2018). Fracture properties of hybrid woven bamboo/woven e-glass fiber composites. Int. J. Struct. Integr..

[88] Shanmugam D., Thiruchitrambalam M., Thirumurugan R. (2014). Continuous unidirectional palmyra palm leaf stalk fiber/glass-Polyester composites: Static and dynamic mechanical properties. J. Reinf. Plast. Compos..

[89] EL-Wazery M.S., EL-Kelity A.M., Elsad R.A. (2020). Effect of water absorption on the tensile characteristics of natural/synthetic fabrics reinforced hybrid composites. Int. J. Eng..

[90] Radif Z.S., Alil A., Abdan K. (2011). Development of a green combat armour from rame-kevlar-polyester composite. Pertanika J. Soc. Sci. Humanit..

[91] Gupta A., Vaishya R., Ahmad Khan K.L., Walia R.S., Singh H. (2019). Multi-response optimization of hybrid filler composition for pultruded jute fiber reinforced polymer composite. Mater. Res. Express.

[92] Onukwuli O.D., Ezeh E.M. (2021). Assessment of the fire retardant effect potential of carbonized cow horn ash additive in Banana peduncle fibre reinforced polyester composites. World J. Eng..

[93] Abdel-Hakim A., Awad E.H., El-Nemr K.F., El-Basheer T.M. (2021). Impact of gamma radiation and multi-walled carbon nanotubes on the mechanical and acoustical properties of reinforced sisal fiber/polyester resin composites. Radiat. Phys. Chem..

[94] Gnaniar K., Kumaran T., Aslan M., Mayandi K. (2020). Mechanical properties of waste copper slag filled surface activated jute fiber reinforced composite. Mater. Res. Express.

[95] Biswas B., Chabri S., Mitra B.C., Das K., Bandyopadhyay N.R., Sinha A. (2017). Effect of copper/graphite addition on electrical conductivity and thermal insulation of unsaturated polyester/jute composites. J. Inst. Eng. India Ser. D.

[96] Arumugam C., Arumugam G.S., Ganesan A., Muthusamy S. (2021). Mechanical and water absorption properties of short banana fiber/unsaturated polyester/molecular sieves + ZnO nanorod hybrid nanobiocomposites. ACS Omega.

[97] Movva M., Kommineni R. (2019). Effect of green gram husk nanocellulose on banana fiber composite. J. Nat. Fibers.

[98] Bakhori S.N.M., Hassan M.Z., Bakhori N.M., Rashedi A., Mohammad R., Md Daud M.Y., Aziz S.A., Ramlie F., Kumar A., Naveen J. (2022). Mechanical properties of PALF/Kevlar-reinforced unsaturated polyester hybrid composite laminates. Polymers.

[99] Zeleke Y., Feleke T., Tegegn W., Atinaf Y. (2022). Design and development of false ceiling board composite material using pineapple leaf Fibre reinforcement in unsaturated polyester matrix. Int. J. Sust. Eng..

[100] Kowshik S., Sharma S., Rao S.U., Shettar M., Hiremath P., Upadhyaya A. (2022). Investigation on the effects of uncarbonised, carbonised and hybrid eggshell filler addition on the mechanical properties of glass fibre/polyester composites. Eng. Sci..

[101] Ahmad Nadzri S.N.Z., Md Shah A.U., Sultan M.T.H., Safri S.N.A., Shahar F.S., Basri A.A. (2022). Failure mechanisms of kenaf/glass sandwich laminates subjected to low velocity impact loading. J. Ind. Text..

[102] Gortner F., Schüffler A., Fischer-Schuch J., Mitschang P. (2022). Use of bio-based and renewable materials for sheet molding compounds (SMC)–Mechanical properties and susceptibility to fungal decay. Compos. Part C.

[103] Mehta G., Mohanty A.K., Thayer K., Misra M., Drzal L.T. (2005). Novel biocomposites sheet molding compounds for low cost housing panel applications. J. Polym. Environ..

[104] Patel H.K., Ren G., Hogg P.J., Peijs T. (2010). Hemp fibre as alternative to glass fibre in sheet moulding compound Part 1-Influence of fibre content and surface treatment on mechanical properties. Plast. Rubber Compos..

[105] Mir Md S.S., Chan M.Y., Koay S.C. (2021). Mechanical properties of polyester/corn husk fibre composite produced using vacuum infusion technique. Polym. Polym. Compos..

[106] Dhakal H., Ghasemnejad H., Zhang Z., Ismail S.O., Arumugam V. (2019). The post-impact response of flax/UP composite laminates under low velocity impact loading. Int. J. Damage Mech..

[107] Rajulu A.V., Devi L., Babu Rao G., Lakshminarayana Reddy R. (2003). Chemical resistance and tensile properties of epoxy/unsaturated polyester blend coated bamboo fibers. J. Reinf. Plast. Compos..

[108] Norlin N., Akil H.M., Ishak Z.M., Abu Bakar A. (2010). Degradation of compressive properties of pultruded kenaf fiber reinforced composites after immersion in various solutions. Mater. Des..

[109] Wong K.J., Khoo S., Low K.O. (2016). Influence of alkali treatment on the interfacial properties of bamboo/polyester composites. Recent. Pat. Mech. Eng..

[110] Guhanathan S., Devi M. (2004). Studies on interface in polyester/fly-ash particulate composites. Compos. Interfaces.

[111] Mwaikambo L., Bisanda E. (1999). Performance of cotton-kapok fabric-polyester composites. Polym. Test..

[112] Kumar R.N., Wei L.M., Rozman H.D., Abusamah A. (1997). Fire resistant sheet moulding composites from hybrid reinforcements of oil palm-fibres and glass fibre. Int. J. Polym. Mater..

[113] Gao C., Wan Y., He F., Liang H., Luo H., Han J. (2011). Mechanical, moisture absorption, and photodegradation behaviors of bacterial cellulose nanofiber-reinforced unsaturated polyester composites. Adv. Polym. Technol..

[114] Uz Zaman S., Shahid S., Shaker K., Nawab Y., Ahmad S., Umair M. (2022). Development and characterization of chemical and fire resistant jute/unsaturated polyester composites. J. Text. Inst..

[115] Alaseel B.H., Nainar M.A.M., Nordin N.A., Yahya Z., Abdul Rahim M.N. (2022). Effect of water absorption on flexural properties of kenaf/glass fibres reinforced unsaturated polyester hybrid composites rod. Pertanika J. Sci. Technol..

[116] Pramudi G., Raharjo W.W., Ariawan D. (2021). Investigation of flexural strength of sandwich panels recycled carbon fibre/polyester with cotton mesh fabric reinforcement in polyurethane core. J. Appl. Polym. Sci..

[117] Binoj J.S., Edwin Raj R., Daniel B.S.S. (2017). Comprehensive characterization of industrially discarded fruit fiber, Tamarindus indica L. as a potential eco-friendly bio-reinforcement for polymer composite. J. Clean Prod..

[118] Valvez S., Maceiras A., Santos P., Reis P.N.B. (2021). Olive stones as filler for polymer-based composites: A review. Materials.

[119] Mujtaba M., Fernandes Fraceto L., Fazeli M., Mukherjee S., Maira Savassa S., Araujo de Medeiros G., do Espírito Santo Pereira A., Mancini S.D., Lipponen J., Vilaplana F. (2023). Lignocellulosic biomass from agricultural waste to the circular economy: A review with focus on biofuels, biocomposites and bioplastics. J. Clean Prod..

[120] Gonçalves F.A.M.M., Fonseca A.C., Cordeiro R., Piedade A.P., Faneca H., Serra A., Coelho J.F.J. (2022). Fabrication of 3D scaffolds based on fully biobased unsaturated polyester resins by microstereo-lithography. Biomed. Mater..

[121] Adil S., Kumar B., Panicker P.S., Pham D.H., Kim J. (2023). High-performance green composites made by cellulose long filament-reinforced vanillin epoxy resin. Polym. Test..

[122] Rafiee K., Kaur G., Brar S.K. (2021). Fungal biocomposites: How process engineering affects composition and properties?. Bioresour. Technol. Rep..

